# Teachers’ perceptions of teaching physical education using online learning during the COVID-19: A quantitative study in Turkey

**DOI:** 10.1371/journal.pone.0269377

**Published:** 2022-06-22

**Authors:** Ferman Konukman, Bijen Filiz, Hüseyin Ünlü

**Affiliations:** 1 Department of Physical Education, College of Education, Qatar University, Doha, Qatar; 2 Department of Coaching Education, Faculty of Sport Sciences, Afyon Kocatepe University, Afyon, Turkey; 3 Department of Physical Education, Faculty of Sport Sciences, Aksaray University, Aksaray, Turkey; St John’s University, UNITED KINGDOM

## Abstract

**Introduction:**

This quantitative study aimed to determine physical education teachers’ (PETs) perceptions of online physical education (OLPE) during the COVID-19 pandemic in Turkey.

**Methods:**

An OLPE teaching survey during the COVID-19 was used, comprising PETs’ opinions on OLPE teaching as well as the advantages, disadvantages, difficulties, and suggestions for OLPE teaching.

**Results:**

A significant difference by gender was identified in the individual approach to student learning, greater student independence, and ongoing monitoring of student results for teachers, parents, and students. In addition, a significant difference was found by gender in the difficulty in implementing the core curriculum content as well as by school type in the lack of proper home equipment, absence of or limited Internet access, and problems with connecting the computer, tablet, or smartphone to the Internet. Moreover, PETs stated that OLPE teaching is not only the best way to transfer basic information, but it also gives them an opportunity to learn digital technology by devoting time for research for self-improvement. However, they stated that students do not perceive OLPE as a lesson, as student participation is lacking. Moreover, there are deficiencies in students’ social-emotional development. They also stated that the content of the lessons was insufficient, and they were unable to make the lesson interesting.

**Conclusion:**

PETs’ suggested recommendations included enriching the course content, increasing the lesson time, developing a new curriculum, and creating a platform to access course resources.

## Introduction

The Coronavirus disease (COVID-19), which was declared a pandemic by the World Health Organization (WHO) on 11 March 2020 [1], has brought about significant changes in the daily lives of people in worldwide. Therefore, COVID-19 is not only the biggest public health threat in recent history, but it has also caused crises in health, economic, educational, social, and political systems, globally.

Although different vaccines have been developed, it is still stated that the most effective way to protect from COVID-19 is social distancing [2]. Maintaining social distancing significantly limited people’s contact with each other, and the education sector was most severely affected by this situation [3]. The closure of educational institutions on April 20, 2020 has affected the educational status of 91.3% (1.575.270.054) of the world’s students, and the number includes university students [4]. In this process, schools in Turkey were initially closed from March 16, 2020 until April 30, 2020, and it was decided to continue education through the TV Channels and Educational Information Network (EBA) within the scope of open and distance education practices at primary and secondary education levels [5]. Due to the increasing number of cases, the closing period was extended and students could not go to school in the spring term and education continued with distance education.

Overall the world most of the education systems delivery of education has been changed [6] and online education is the most popular distance education way was choosing to deliver lessons during this period [7]. Even before Covid 19, in the education system transferring and conducting the lessons from face-to-face to the online platform has been an important subject for a long time [8, 9].

Physical education (PE) is a school subject (K-12) that has undergone major changes due to improvements in digital technology, owing to COVID-19. These developments have resulted in the shift in PE from face-to-face to hybrid and online physical education (OLPE) [10]. However, OLPE faces problems similar to those of other content areas in terms of academic integrity, student preparedness, motivation, student retention, and technological issues. OLPE’s challenges include teaching motor skills, sports skills, dance, and fitness, and ensuring social-emotional development. Therefore, meeting some of the goals of PE with OLPE becomes extremely difficult. In addition, questions and concerns are raised regarding whether physical education teachers (PETs) are equipped to meet the needs of students, while adapting to this new learning environment. Despite these challenges, teachers should provide information on student performance. When students receive feedback, they understand their level of performance of skills [11, 12].

Initial awareness of the K-12 OLPE was brought about by the Society of Health and Physical Educators of the Nation report, which was co-authored by the American Heart Association and the Society of Health and Physical Education (SHAPE), America [13]. As a result, some deficiencies in OLPE teaching were identified. Thus, SHAPE America [13] suggested that OLPE should proceed with a blended model. The OLPE hybrid method is student-centered; moreover, a majority of the course work takes place outside the classroom, and assessment includes periodic face-to-face meetings for instruction and safety guidelines [13, 14]. SHAPE America [13] then published and supported OLPE standards. The most common form of OLPE is a physical fitness elective course, commonly known as “health for life” [15], and its primary purpose is to improve health behaviors and fitness levels. In addition, the aim of school physical education is to “develop physically literate individuals who have the knowledge, skills, and confidence to enjoy lifelong healthy physical activity” [16].

While there are several curriculum models for face-to-face PE, there is a lack of understanding regarding the course requirements of hybrid and OLPE teaching [17, 18]; moreover, as OLPE is fairly recent, it lacks established curriculum models. As the OLPE curriculum primarily focused on cognitive aspects, it comprised minimal physical activity [19]. Daum and Buschner [19] revealed the lack of motor skill development and limited student participation in a high school OLPE course in their study. They also stated that less than 30% of the teachers were able to complete the recommended 225 minutes of PE. PETs stated that limited technological skills prevented students from completing the course. Furthermore, Corbin and Le Masurier [20] determined that 67% of middle school OLPE teachers followed the "Fitness for life" curriculum, which was part of a secondary school PE textbook. Regarding evaluation, the most common form of assessment in OLPE courses includes physical activity diaries to assess physical fitness and activity levels, and tests or examinations to assess cognitive learning [19, 21].

Moreover, while PETs’ ability to meet student needs in OLPE lessons is an issue that is worth examining, limited studies have been conducted on this subject [19, 21–24]. According to these studies, due to the uncertainty of the curriculum and an unclear application method, PETs have several concerns regarding OLPE teaching; they include course contents, access to students, consideration for individual differences, efficiency, equipment adequacy, and limited Internet access.

It is important to determine whether PETs meet the needs of students in OLPE courses, in addition to qualified education. In addition, in OLPE teaching, teachers experience challenges in transferring traditional movement-oriented course content [25] and student experiences to a virtual environment. Moreover, Turkish PETs’ concerns are also a result of the lack of clarity regarding the instructional method of OLPE in the current curriculum. Thus, given the various instructional variables of PE, it is important for PETs to understand the potential barriers, benefits, and online student outcomes of OLPE teaching, realistically. Further, given the ongoing pandemic, OLPE teaching in Turkey is a new concept for PETs. While evaluations of OLPE teaching have been conducted in different countries for different purposes, there is no evidence on OLPE teaching in Turkey. Therefore, this study aimed to determine PETs’ perceptions regarding the advantages, disadvantages, difficulties, concerns, and suggestions for OLPE teaching, while comparing them based on gender and school type in Turkey. The findings of this research are expected to contribute to knowledge and policy-level changes for OLPE teaching in Turkey.

## Method

### Research design

This study used a quantitative design to determine Turkish PETs’ opinions on OLPE teaching during the COVID-19 pandemic. A quantitative research design facilitates observations by objectifying experienced events and phenomena through numerical expressions of measurements [26]. The study consisted of PETs’ opinions on OLPE teaching during COVID-19, including its advantages, disadvantages, the difficulties experienced, and suggestions for OLPE teaching during the pandemic. Some items of a questionnaire were used for quantitative design. In addition, the answers received from the items were supported by open-ended questions.

### Participants

The study was conducted during the 2019–2020 spring academic semester. The participating PETs were working in different regions and provinces. The population of PET in Turkey is 35,000. A total of 335 PETs participated in this study, voluntarily. Of the participants, 154 (46.0%) were female and 181 (54.0%) were male. Furthermore, 283 (85.5%) and 52 (15.5%) PETs worked at a public school and private school, respectively. Moreover, 169 (50.4%) PETs worked at a secondary school, while 166 (49.6%) worked at a high school. Moreover, participants had average work experience of 15.87 (SD = 8.63) years as PETs.

### Procedure

The study was granted ethical approval by the ethics committee of Afyon Kocatepe University (Approval number: 09, 12.10.20). Participation in the study was voluntary. In addition, informed consent was obtained from all participants.

PETs were recruited using an open sampling procedure [27], which is a convenience sampling technique, as anyone who met the inclusion criteria and expressed interest were included in the analysis. An electronic questionnaire was sent to those who wished to participate in the study using e-mail, personal media, and social media accounts. Prior to the study onset, a consent form was provided along with the survey, which entailed the purpose of the study, time allocation, inclusion criteria, and the right to withdraw from participation. The inclusion criteria were as follows: PETs working in a private or public school as well as those working in a secondary or high school. PETs who met the inclusion criteria completed the survey, which included four items and open-ended questions each.

### Measures

In this study, some items of the OLPE Teaching Survey During the COVID-19, developed by Korcz et al. [28] were used. Prior to inclusion, due permission was obtained from the researchers. The survey consisted of two sections: (1) demographic information and (2) subjective assessment of online teaching in PE in the times of the COVID-19 pandemic. The second part of the survey comprised of 25 questions and included single- and multiple-choice questions. To evaluate the quality of implementation and PE teachers’ perceived advantages, disadvantages, and encountered difficulties of online PE teaching during the first wave of the COVID-19 pandemic, only selected questions (only four items and four open-ended questions) from the survey were used in this study. The survey was translated into Turkish, after which it was submitted to three experts for content validity [26]. To assess the validity of the survey, the agreement level of expert opinions was calculated to be 89%. Further, it was revised in accordance with these suggestions, after which it was administered to 30 PETs, resulting in reception of positive feedback. In the final form, four items, including the advantages, disadvantages, difficulties, and concerns of OLPE teaching, had to be rated using three response options: very important (3), important (2), and less important (1). In addition, four open-ended questions included PETs’ own opinions regarding the advantages, disadvantages, difficulties, and suggestions for OLPE teaching.

### Data analysis

Data analysis began with standard procedures for data cleaning and screening [29]. No data were extracted from the dataset because of the lack of extreme values that would affect the data analysis. Frequency and percentage analyses were used to determine the demographic variables, while chi-square analysis was used to determine the opinions of PETs on OLPE teaching by gender and school type. In addition, in order to support quantitative design, inductive content analysis was used for PETs’ responses to open-ended questions. Members of the research utilized inductive content analysis and constant comparison [30] to identify initial categories and emergent themes from the data [27]. Initial categories were discussed among researchers to explore the similarities and differences among the groups until an agreement about the coding protocol was established and verified. The second cycle coding employed a thematic analysis. Quotes from the data were extracted that reflect the most essential and distinctive aspects of the OLPE’s own opinions. Marton [31] described the process as having “quotes sorted into piles, borderline cases are examined, and eventually the criterion attributes for each group are made explicit”. For the inductive content analysis, coding of data, theme identification, organization of the data according to the theme codes, and interpretation of the findings were followed. The data set was coded as “PET 1, PET 2, and PET 3….” As a result, six-page long answers were obtained from the open-ended questions, along with 295 coded units. Finally, for an internal measure of reliability, the agreement between the two researchers was calculated by “consensus,” as suggested by Miles et al. [32]. A consensus of 88% was attained from the data obtained from open-ended questions.

## Results

### Advantages of online physical education teaching

PETs were required to rank the three most important advantages of OLPE teaching. Table 1 shows the results.

**Table 1:** Pets’ opinions on the advantages of online physical education teaching by gender.

	Gender	Less important n (%)	Important n (%)	Very important n (%)	df	χ2	P
The possibility of using modern technologies in practice	Female	25 (16,2%)	61 (39,6%)	68 (44,2%)	2	4.623	.099
	Male	41 (22,7%)	53 (29,3%)	87 (48,1%)			
Individual approach to student learning	Female	22 (14,3%)	78 (50,6%)	54 (35,1%)	2	11.609	.009*
	Male	51 (28,2%)	65 (35,9%)	65 (35,9%)			
Greater student independence	Female	51 (33,1%)	63 (40,9%)	40 (26,0%)	2	10.232	.006*
	Male	37 (20,4%)	71 (39,2%)	73 (40,3%)			
Better relationships with students	Female	61 (39,6%)	38 (24,7%)	55 (35,7%)	2	.604	.739
	Male	66 (36,5%)	51 (28,2%)	64 (35,4%)			
Easier implementation of the core curriculum content (e.g., related to health education)	Female	32 (20,8%)	56 (36,4%)	66 (42,9%)	2	1.218	.544
	Male	45 (24,9%)	68 (37,6%)	68 (37,6%)			
The possibility of using interesting forms of technology to assist students in learning	Female	24 (15,6%)	64 (41,6%)	66 (42,9%)	2	1.186	.553
	Male	31 (17,1%)	67 (37,0%)	83 (45,9%)			
An attractive and effective form of material checking	Female	49 (31,8%)	56 (36,4%)	49 (31,8%)	2	2.019	.364
	Male	70 (38,7%)	55 (30,4%)	56 (30,9%)			
Ongoing monitoring of student results for teachers, parents, and students	Female	17 (11,0%)	73 (47,4%)	64 (41,6%)	2	26.445	.000*
	Male	58 (32,0%)	48 (26,5%)	65 (41,4%)			
An attractive way of presenting my competencies to students	Female	33 (21,4%)	71 (46,1%)	50 (32,5%)	2	.734	.693
	Male	46 (25,4%)	79 (43,6%)	56 (30,9%)			

*p < .01

In Table 1, in PETs’ opinions regarding the advantages of OLPE teaching, a significant difference was observed in the individual approach to student learning, greater student independence_,_ ongoing monitoring of student results for teachers, parents, and students by gender. According to the results, 85.7% of female PETs and 71.8% of male PETs stated that the individual approach to student learning were important and very important advantages, respectively. Moreover, 66.9% of female PETs and 79.5% of male PETs stated that greater student independence were important and very important. Regarding the ongoing monitoring of student results for teachers, parents, and students, 89.0% of female PETs and 67.9% of male PETs stated that they are important and very important. For other variables, no significant differences were found by gender (p>.01).

### Disadvantages of online physical education teaching

PETs were asked to rank the three most important disadvantages of OLPE. The results are shown in Table 2.

**Table 2:** Pets’ opinions on the disadvantages of online physical education teaching by gender.

	Gender	Less important n (%)	Important n (%)	Very important n (%)	df	χ2	p
Limited contact with students	Female	37 (24,0%)	42 (27,3%)	75 (48,7%)	2	.950	.622
	Male	36 (19,9%)	55 (30,4%)	90 (49,7%)			
Inability to verify the implementation of movement tasks in an appropriate way	Female	33 (21,4%)	45 (29,2%)	76 (49,4%)	2	2.735	.255
	Male	46 (25,4%)	39(21,5%)	96 (53,0%)			
Inability to monitor student progress in a satisfactory way	Female	38 (24,7%)	49 (31,8%)	67 (43,5%)	2	1.157	.561
	Male	37 (20,4%)	56 (30,9%)	88 (48,6%)			
Lack of a personalized approach with students	Female	30 (19,5%)	48 (31,2%)	76 (49,4%)	2	2.959	.228
	Male	23 (12,7%)	64 (35,4%)	94 (51,9%)			
Difficulty experienced with implementation of the core curriculum content (e.g., related to the area of physical development and physical fitness)	Female	36 (23,4%)	48 (31,2%)	70 (45,5%)	2	6.248	.044*
	Male	29 (16,0%)	79 (43,6%)	73 (40,3%)			
More theories and less practice	Female	32 (20,8%)	47 (30,5%)	75 (48,7%)	2	.768	.681
	Male	32 (17,7%)	53 (29,3%)	96 (53,0%)			
Difficulty with motivating students to learn and work independently	Female	24 (15,6%)	67 (43,5%)	63 (40,9%)	2	4.707	.095
	Male	20 (11,0%)	66 (36,5%)	95 (52,5%)			

*p < .01

In Table 2, a significant difference was observed regarding PETs’ perceptions of the disadvantages of OLPE teaching by gender in difficulty experienced with implementation of the core curriculum content. Overall, 76.7% of female PETs and 83.9% of male PETs stated that the difficulty experienced with implementation of the core curriculum content were important and very important. For other variables, no significant differences were found by gender (p> .01).

### Difficulties experienced during online physical education teaching

Table 3 shows the results of PETs’ three most important difficulties of OLPE teaching.

**Table 3:** Pets’ opinions on the difficulties of online physical education teaching by school type.

	School type	Less important n (%)	Important n (%)	Very important n (%)	df	χ2	p
Lack of proper equipment at home (e.g., laptop, tablet, speakers, headphones, and microphone)	State	69 (24,4%)	89 (30,7%)	127 (44,9%)	2	7.883	.019*
	Private	12 (23,1%)	7 (13,5%)	33 (63,5%)			
Lack of proper training in using technology	State	68 (24,0%)	114 (40,3%)	101 (35,7%)	2	1.174	.556
	Private	9 (17,3%)	22 (42,3%)	21 (40,4%)			
Lack of experience with applications or platforms that can be used for online learning	State	65 (23,0%)	127 (44,9%)	91 (32,2%)	2	1.810	.404
	Private	16 (30,8%)	19 (36,5%)	17 (32,7%)			
Lack of support from the management	State	98 (34,6%)	93 (32,9%)	92 (32,5%)	2	2.612	.271
	Private	24 (46,2%)	15 (28,8%)	13 (25,0%)			
Absence of or limited Internet access	State	55 (19,4%)	91 (32,2%)	137 (48,4%)	2	10.574	.005*
	Private	14 (26,9%)	19 (36,5%)	19 (36,5%)			
Problems connecting the computer, tablet, or smartphone to the Internet	State	57 (20,1%)	87 (30,7%)	139 (49,1%)	2	34.414	.000*
	Private	18 (34,6%)	4 (7,7%)	30 (57,7%)			
Lack of support from colleagues	State	87 (30,7%)	96 (33,9%)	100 (35,3%)	2	1.936	.380
	Private	21 (40,4%)	16 (30,8%)	15 (28,8%)			
Large class size	State	91 (32,2%)	104 (36,7%)	88 (31,1%)	2	3.793	.150
	Private	19 (36,5%)	12 (23,1%)	21 (40,4%)			

*p < .01

Table 3 shows that a significant difference was found regarding PETs’ opinions on the difficulties of OLPE teaching by school type in lack of proper home equipment, absence of or limited Internet access, and problems with connecting the computer, tablet, or smartphone to the Internet. The results indicated that 44.9% of PETs working in public schools and 63.5% of those working in private schools stated that the absence of proper home equipment is a very important difficulty of OLPE teaching. Further, 48.4% of PETs working in public schools and 36.5% of those working in private schools stated that the absence of or limited Internet access was a very important difficulty of OLPE teaching. Overall, 49.1% of PETs working in the state schools and 57.7% of those working in private schools stated that problems related to connecting the computer, tablet, or smartphone to the Internet were very important difficulties. For the other variables, no significant differences were found by school type (p> .01).

### Concerns regarding online physical education teaching

Table 4 shows the concerns of PETs regarding OLPE teaching. A significant difference was found in these concerns by gender. The results indicated that 35.8% of PETs were not worried, 19.7% of PETs were worried about student safety, and 7.2% were concerned that their image would be used for non-essential or non-didactic purposes. Furthermore, 11.9% of PETs were worried about the difficulties related to the use of Internet applications, while 3.3% of PETs were concerned about making errors during recording or transmission. Finally, 22.1% of PETs expressed concern about the visibility of materials, which were their intellectual property, on the Internet, without their consent and knowledge.

**Table 4:** Pets’ opinions on the concerns about online physical education teaching by gender.

	Variables	Male n (%)	Female n (%)	Total	df	χ2	p
Concerns about OLPE^a^ teaching	I have no worries	63 (18.8%)	57 (17.0%)	120 (35,8%)	5	20.646	.001*
	Student safety (e.g., injury, accidents)	29 (8.7%)	37 (11.0%)	66 (19.7%)			
	My image will be used for non-essential/ non- didactic purposes	8 (2.4%)	16 (4.8%)	24 (7.2%)			
	Using internet applications is difficult	12 (3.6%)	28 (8.4%)	40 (11.9%)			
	Making errors during the recording or transmission,	0 (0.0%)	11 (3.3%)	11 (3.3%)			
	Materials that are my intellectual property will be visible on the internet without my consent and knowledge	42 (12.5%)	32 (9.6%)	74 (22,1%)			

*p < .01

^a^OLPE = online physical education

### Physical education teachers’ opinions on online physical education teaching

Table 5 shows the result of PETs disadvantages, difficulties, and suggestions for OLPE teaching, in addition to the content analysis.

**Table 5:** Pets’ opinions on online physical education teaching.

Themes	Sub-themes	Frequency (f)	Percent (%)
Advantages	Conveying basic knowledge	48	14.3
	Learning digital technology	40	11.9
	Conducting more research for self-improvement	22	6.6
	Devoting more time to OLPE^a^ teaching	34	10.1
Disadvantages	Students did not perceive PE^b^ as a course	43	12.8
	Most students did not participate in PE^b^ lessons	62	18.5
	Deficiencies in the socio-emotional development of students	34	10.1
Difficulties	OLPE^a^ course content was inefficient	85	25.4
	Unable to make the course interesting	33	9.9
Suggestions	Course content should be enriched	146	43.6
	Lesson time should be increased	27	8.1
	A new curriculum for OLPE^a^ teaching should be developed	32	9.6
	A platform to access course resources should be created	53	15.8

^a^OLPE = online physical education

^b^PE = Physical education

When asked whether they had other opinions about the advantages of OLPE teaching, PETs stated that OLPE teaching was the best way to convey basic knowledge (n = 48, 14.3%). For example, PET 13, 99, 121, and 279 stated, “*The lessons were mostly practice-based*, *and we did not give much theoretical knowledge*. *Online education was the best and easiest way to transfer theoretical knowledge*.” Further, PET 58 stated, “*The students did not want to sit in the classroom and get theoretical information in the PE class*, *which is two hours a week*. *They wanted to move and engage in physical activities*. *We had difficulties in providing theoretical information*. *In this respect*, *online education has provided us with the expression*.”

As an advantage, PETs stated that they had the opportunity to learn and better their understanding of digital technology (n = 40, 11.9%). For example, PET 44 and PET 305 stated, “*I did not use the computer unnecessarily*. *Thanks to this*, *I had to learn online applications*.” Further, PET 287 and PET 312 stated, “*I am advanced*, *I don’t understand much about technology*, *I started using technology thanks to online education*.”

As an advantage, PETs stated that they could conduct research for self-improvement (n = 22, 6.6%). For example, PET 110 stated, “*I have the chance to do more research since we work from home*, *I look for various resources to diversify the lessons and be useful to the students…I use them in online lessons*.” PET 234 stated, “*We were always practicing the lessons*, *we did not have too many resources on theoretical knowledge*, *now because I have time*, *I do research and increase my course resources*.”

Finally, PETs stated that they could devote more time to OLPE teaching (n = 34, 10.1%). For example, PET 59 and PET 278 stated, “[As] *we were spending a lot of time at school*, *we did not have much time for ourselves and our family…now we have more time for ourselves*.” PET 333 stated, “*I was spending most of my time in school*, *now I can devote more time to myself*, *both* [for] *resting and researching…I can have a good time with my family*.”

PETs were asked whether they had other opinions about the disadvantages of OLPE teaching. However, PETs stated that students did not perceive PE as a course (n = 43, 12.8%). For example, PET 14, PET 120, and PET 253 stated, “*Students do not take the course seriously*.” Moreover, PET 23 and PET 304 stated, “*We generally provide theoretical information in online education*. *Because students consider the course as a physical activity*, *they do not participate in online courses*.” PET 142 stated, “*Students care more about other courses*, *this course was already seen as an exam preparation course*, *now it is perceived as an empty course*.” Furthermore, PET 259 stated, “*Students think that they will pass this course with high marks already*, *this perception should be broken and students should be given the perception that this course is as important as other academic courses*.”

Moreover, as a disadvantage, PETs stated that most students did not participate in PE lessons (n = 62, 18.5%). For example, PET 162 stated, “*Most students do not attend the class because there is no attendance requirement*, *although I do not write from the group*, *the attendance does not exceed 3–5 students*.” PET 89 stated, “*Some of the students did not have a computer*, *and some of them had problems with internet connection*. *Families also do not care much about the lessons; there are also families who use their children to help in daily work*. *In addition*, *students do not perceive the activities as a lesson* [that] *they have to attend*. *Therefore*, *most of the students did not attend class*.”

In addition, some PETs stated that deficiencies in the socio-emotional development of students, owing to the pandemic, act as a disadvantage (n = 34, 10.1%). For example, PET 279 stated, “*Students at school were experiencing feelings of gain*, *loss*, *empathy*, *excitement*, *and sadness in lessons*. *Now* [that] *they are locked in homes*, *they cannot experience these feelings with distance lessons*.” PET 159 stated, “*Students used to communicate at school*, *imitate each other*, *learn from each other; now they are left alone at home*, *they cannot socialize and experience their emotions*.”

PETs were asked if they had other opinions regarding the difficulties of OLPE teaching. The PETs stated that the OLPE course content was inefficient (n = 85, 25.4%). For example, PET 94 stated, “*We cannot diversify the course for online education*, *sufficiently*. *In addition*, *physical activities on Education Information Network TV (EBA TV) are also insufficient*.” Moreover, PET 44 stated, “*Unfortunately*, *I do not have enough resources*, *it takes a lot of time to plan a course* [that is] *suitable for every grade and it may not always be efficient*.”

Some PETs stated that it was difficult for them to make the course interesting (n = 33, 9.9%). For example, PET 56 stated, “*When the lesson is not interesting*, *the student leaves the lesson and enters another lesson… we cannot keep the student in the lesson*. *The activities on EBA TV do not interest them either*.” Furthermore, PET 105 stated “*I use various materials in the course*, [including] *games*, *puzzles*, *technical information*, *etc*. *I am preparing*, *but it does not interest the student*.’

PETs were asked about their suggestions for OLPE teaching. The PETs stated that the course content should be enriched (n = 146, 43.6%). For example, PET 165 stated, “*Especially the activities on EBA TV should be organized not only in the morning but also at the changing times of the day and for each grade level*: *Yoga*, *pilates*, *aerobics*, *games*, *dance*, *etc*.*”* Moreover, PET 293 stated, “*We should create content not only for theoretical knowledge*, *but also for physical activities in our own lessons…we have to make them move*, *and we have to prepare activities that are appropriate for the students’ class levels*.”

PETs suggested that the lesson time should be increased (n = 27, 8.1%). For example, PET 46, PET 99, and PET 123 stated, “*The duration of physical activity in EBA TV is 10–15 minutes*, *which is very short*, *the activity time must be increased and spread throughout the day*.” Further, PET 14 stated, “*Especially the duration of physical activities offered on EBA TV should be increased*. *In online lessons*, [it is] *one day* [in] *a week and one lesson lasts 30 + 30 minutes*. *To be more efficient*, *the day and time of the lesson can be increased*.”

PETs suggested the development of a new curriculum for OLPE teaching (n = 32, 9.6%). For example, PET 119 stated, “*We have difficulties in finding activities for the courses*, [as] *the current curriculum does not fit online education…we cannot design every activity online*, *since the content of the program is generally oriented toward practice*. *An update is required in the curriculum for online education*.” In addition, PET 268 stated, “*We try to maintain online education according to the contents of the current curriculum*, *but we cannot be as effective as in face-to-face education*. *We try to convey the subject with presentations and videos*, *because the students cannot learn by doing and experiencing… what we tell remains abstract*. *For this*, *the content of online education needs to change’*.”

Finally, PETs suggested creating a platform where they could access course resources (n = 53, 15.8%). For example, PET 56 stated, “*We do not have material on every subject*, *we experienced a lot of difficulties in the beginning; everyone was asking each other*, [and] *then some social media groups started sharing*. *It would be nice to institutionalize this and create a platform where various resources for online education related to our course can be shared at every grade level*.” Moreover, PET 312 stated, “*We access the course resources mostly through social media or a few websites*, *but it is not sufficient for the current system*. *I also try to create a course resource myself*, *but this is not enough*. *It would be great if we had a corporate platform like SHAPE America*.”

## Discussion

This study presented PETs’ opinions on the advantages, disadvantages, difficulties, concerns, and suggestions for OLPE teaching.

First, we highlighted PET’s opinions on the advantages of OLPE teaching by gender. A significant difference was determined by gender in the individual approach to student learning, greater student independence, and ongoing monitoring of student results for teachers, parents, and students. According to the results, female PETs considered the individual approach to student learning as an advantage of OLPE, while male PETs considered greater student independence as an advantage. Moreover, female PETs considered the ongoing monitoring of student results for themselves, parents, and students as an advantage of OLPE. Thus, female PETs consider OLPE more appropriate than face-to-face education to facilitate the individual approach to student learning. Daum and Buschner [19] and Williams [22] supported this finding in their studies by reporting that OLPE helps teachers get acquainted with students on a one-to-one basis. In this regard, being able to get to know the students and provide education according to their interests and needs is an advantage of OLPE teaching. However, male PETs consider OLPE teaching as more appropriate for student independence. In this current study, although there was no significant difference, it was determined that the rate of using modern technologies in practice was higher in male PETs than in women PETs. In this context, it is understood that male PETs are able to offer various course content options to students by using technological tools more in practice. Enabling students to make independent programs by setting goals for themselves, developing and evaluating their programs, and raising their awareness regarding the stages of development make significant contributions to lifelong learning [33]. Therefore, OLPE teaching encourages students to conduct independent studies. Also, the fact that male PETs can use technological tools provides an advantage in terms of different course applications.

This study highlighted PETs’ opinions on the disadvantages of OLPE teaching by gender. A significant difference was found by gender in difficulty experienced in the implementation of the core curriculum content. PETs believe that the basic curriculum content is more difficult to apply using OLPE teaching; moreover, male PETs found it to be more disadvantageous, compared with female PETs. Most of the physical education course content is practice-oriented in Turkey. Also, as there is no standard teaching model for OLPE teaching in Turkey, teachers may face challenges in creating course content and presenting a program that covers all standards to students. Thus, the lack of an OLPE teaching model may cause concern and hesitation regarding the course’ implementation and interpretation among PETs. Critics have asserted that the fact that students are neither physically active nor engaged in motor learning is a disadvantage of OLPE [34–36]. Furthermore, Daum and Buschner [19] found that neither did OLPE courses meet the learning standards of students nor did they have sufficient physical activity. In addition, some authors have expressed concerns about the accuracy and accountability of student learning, especially in tracking their physical activity levels [19, 22, 35]. In addition, SHAPE America considers the development of motor skill competence as the highest priority of PE because of its impact on student engagement, intrinsic motivation, perceived competence, participation in physical activity, and adequate health-related fitness levels [16]. Therefore, the current OLPE teaching does not adequately meet these objectives and the implementation of curriculum content.

This study presented PET’s opinions on the difficulties of OLPE teaching by school type. A significant difference was determined by school type in lack of proper home equipment, lack of or limited Internet access, and problems with connecting the computer, tablet, or smartphone to the Internet. A higher proportion of PETs employed in private schools experienced difficulty due to the lack of proper home equipment, while those employed in public schools experienced difficulty due to absence of or limited Internet access. Moreover, a higher proportion of PETs employed in private schools experienced difficulty due to problems related to connecting the computer, tablet, or smartphone to the Internet. Private schools in Turkey, according to the state school course content, are known to be busier due to a greater number of events. In addition, private schools use more technological equipment and materials. Therefore, home equipment and Internet access are important for transferring the systems used in private schools onto an online platform. In Turkey, especially in the Eastern enclave, some students studying in state schools do not have computer and Internet access. Therefore, to conduct online education effectively, The Ministry of National Education (MEB) is expanding its Internet network, establishing internet-computer centers in certain regions, and distributing free tablets to students. However, reports claim that despite these measures, 54% of students still cannot access the EBA TV [37]. Due to the ongoing nature of the pandemic, students are still restricted to their homes; thus, it is essential to solve the problems of computer and internet access. Therefore, SHAPE America [13] has encouraged the search for common technology (i.e., Internet, computer, etc.) for the implementation of OLPE courses and the introduction of a hybrid or blended model to alleviate concerns regarding learning outcomes.

This study highlighted PETs’ opinions regarding OLPE teaching, and found a significant difference by gender. According to the results, it was determined that female PETs were less concerned about OLPE teaching, and more concerned that the materials, which are their intellectual property, were visible on the Internet without their consent and knowledge, compared with male PETs. However, male PETs were more concerned about student safety related to OLPE teaching and that their image could be used for non-essential or non-didactic purposes. Moreover, they were concerned about the difficulties experienced when using internet applications, as well as making errors during the recording or transmission. Thus, female PETs had fewer concerns about OLPE teaching. Moreover, both female and male PETs were concerned about safety for themselves and students while using technology for learning. Similarly, Daum and Buschner [19] and Mosier [35] determined that besides teachers’ positive thoughts on OLPE teaching, they had concerns about safety and responsibility issues for students. In this context, it is considered important that families are informed about safe internet use and that they convey this information to their children.

In this study, PETs were required to provide their opinions regarding the advantages of OLPE teaching. PETs stated that OLPE teaching was the best way to convey basic knowledge. In Turkey, PE lessons at weekly school programs are usually implemented once a week and two hours in a day. Therefore, students wanted to be physically active in the garden or gym during this period. Pre-COVID-19, teachers continued their lessons in a more practical manner, in accordance with the content of the curriculum, with limited inclusion of theoretical knowledge in the lessons. However, it is understood that teachers transferred more theoretical knowledge in OLPE teaching during the pandemic. Therefore, OLPE teaching has become an intermediary platform for the transfer of theoretical knowledge of PE to students. However, some studies [13, 19] found that most of the teachers in OLPE used a curriculum focused on the life-long health-related cognitive level of knowledge transfer. This result is consistent with previous research findings.

In this study, PETs stated that they had the opportunity to learn digital technology better with OLPE teaching. Moreover, PETs in Jannah et al.’s study [38] emphasized that effective digital-based learning depends on teacher competence (i.e., digital skills, creative thinking, and communication skills) rather than the availability of digital facilities (i.e., technological tools or equipment). In Turkey, teachers who are in the process of receiving technology-based courses are provided undergraduate education and in-service training [39]. Rachmadtullah et al. [40] found that during the pandemic, teachers were also affected by their technological readiness for the implementation of online distance education. Therefore, OLPE teaching contributes to teachers’ use and developments in digital technology. However, Williams [22] emphasized that PETs did not receive sufficient training for OLPE. Therefore, it was suggested that PETs who have just started OLPE education should receive education [19]. However, while the OLPE approach for dealing with training for PETs in Turkey is thought to be essential, it is not yet available.

PETs stated that they could do more research and devote more time for self-improvement during the OLPE teaching process. Thus, PETs continued their OLPE education, owing to flexible working from home hours during the pandemic. They spent time on self-improvement by researching and focusing on themselves and their families. Williams [22], in his study on OLPE, found that students liked OLPE courses because of the flexibility of the program, the choice of physical activity, and the opportunity to exercise in a comfortable environment. They also appreciated that teachers also enjoyed the flexibility of the program. This result is consistent with the results of previous studies.

In this study, PETs were asked whether they had other opinions about the disadvantages of OLPE teaching. PETs stated that the students did not perceive the course as an important one, resulting in low student attendance. PETs stated that the physical activity content on EBA TV was insufficient, and that most students did not prefer to participate in these activities. EBA TV is an alternative course platform developed by the MEB for distance education. It has videos and documents prepared for K-12 and all kindergarten classes. Thus, students who lack Internet access or computers generally prefer this platform. Moreover, İpek et al. [41] examined the problems and solution suggestions for distance learning during COVID-19, and revealed that 60% of the students did not log onto EBA TV. This result indicates that only approximately 60% students had Internet access and technological means in Turkey. Teachers conducted their online education remotely using the EBA platform, according to their school schedule; teachers uploaded their documents onto the EBA, while students with Internet access and technological devices (e.g., computers, phones, tablets, and laptops) preferred following their school teachers’ lessons. Since the conditions across the regions of Turkey are similar, in terms of Internet access, material, etc., there is no obligation to follow the course for the students. Therefore, in this context, teacher competencies come to the fore. Rachmadtullah et al. [40] pointed out that during the pandemic, student engagement, interest, and motivation decreased or disappeared over time; thus, teacher’s equipment is crucial in preventing this situation.

PETs highlighted the deficiencies in the socio-emotional development of students in OLPE teaching. The COVID-19 pandemic not only threatens school-age children but also individuals of all age groups, thus preventing children from engaging in their social life due to restrictions on going to school, playing on the streets, shopping in malls, using sports centers, etc. Thus, teachers’ efforts to enhance the socio-emotional development of children within the scope of OLPE teaching are important [42]. As it can be difficult to address the socio-emotional aspects of PE curricula without interaction [19], it is important to design activities that enhance student interaction within OLPE. One area that draws much attention is the use of exergames in PE classes. The use of competitive or collaborative exergames in an OLPE course can not only benefit students physically and socially, but can also help fill the socio-emotional curriculum gap [23, 43].

This study highlighted PETs’ opinions on the difficulties of OLPE teaching. PETs stated that the course content was inefficient; moreover they were also unable to make the course interesting. As there is little empirical evidence to help students effectively acquire PE knowledge and skills remotely [13], online education is believed to reduce students’ interest in the lesson. Rachmadtullah et al. [40] found that teachers were most affected by changes in instructional strategies and techniques during the pandemic. Furthermore, this study also emphasized that teachers’ online (e.g., TV, radio, digital applications, etc.) or offline (e.g., written instructional documents, textbooks, etc.) technological capacity significantly contributes to successful learning. In this context, teachers are required to prepare efficient and engaging lecture presentations by blending online and offline resources to enhance student participation in the lesson. It is also important for teachers to not lose their motivation to engage students in these activities. During the COVID-19 pandemic, MEB should provide the necessary support to prevent teachers from exhausting and losing their motivation in the process of adapting to these changes.

Finally, PETs were asked to provide suggestions for OLPE teaching. PETs suggested that enriching the OLPE content by creating a platform through which they could access various course-related resources is warranted. In Turkey, PETs access course resources using several websites. However, there is no enterprise-level platform. Similar to SHAPE America [44] that published "Guidelines for Online Physical Education," Turkey should also create such a platform for teachers.

PETs suggested that increasing the lesson time in OLPE teaching is essential, especially on EBA TV. Moreover, they stated that the duration of physical activity is insufficient, as each grade has PE classes only once a week in weekly school programs. A survey of OLPE classes found that approximately 75% of the courses did not meet the national guidelines for weekly physical activity duration [19]. Studies have shown that students perform better academically after a short physical activity (≤ 20 minutes) [45]. Therefore, especially on EBA TV, the duration of physical activity can be increased, spread out throughout the day, or PE activities can be included in the school program on a daily basis.

Moreover, PETs suggested that the development of a new curriculum for OLPE teaching is necessary. Thus, the current PE curriculum should be used to ensure continuity in OLPE, along with a diverse range of instructional approaches [14]. In OLPE, it is very important for teachers to be able to create the best practices in pedagogy, similar to that of a traditional PE environment. In the field of PE, concerns about distance learning include the sedentary nature of distance learning, the lack of social contact, and the inability of teachers to monitor student progress, visually [23]. One of the most important concerns of K-12 OLPE critics is that students are neither physically active nor engaged in motor learning [13, 34–36]. Similarly, studies including activities such as performance tests, journaling, video recording, and virtual field trips in OLPE have been presented [14]. However, most of the authors emphasized that further research is needed to validate K-12 OLPE; moreover, new learning technologies should be aimed at maximizing student learning [34–36]. Therefore, there is a need to develop an evidence-based curriculum that adapts the instructional approach to OLPE [14, 34]. In addition, in the OLPE program, a cognitive, affective, and psychomotor evaluation that considers students’ individual capacities should be developed. Interactive activities can be included in the program to maintain students’ physical fitness levels, increase their pedagogical readiness, and prioritize their social-emotional development [11].

## Conclusions

This study is one of the few studies on PETs in Turkey. This study included only PETs’ advantages, disadvantages, difficulties, concerns, and suggestions regarding OLPE teaching. Future studies can be conducted by reproducing data groups from different countries. Qualitative research can be conducted on teachers’ OLPE teaching experiences. In addition, a versatile broad outline can be produced by considering the opinions of PETs working in different countries to create a new OLPE model. Finally, experimental studies can be conducted on PETs to receive OLPE teaching in PE. The results of this study can help PE curriculum specialists understand possible situations that may arise during the creation of an OLPE model. The results indicated that a new curriculum should be created for instructional approaches to OLPE teaching. Moreover, teachers should be trained, accordingly. For example, establishing an institutional platform to access course resources may facilitate the implementation of PETs. In addition, PETs should be offered in-service seminars on technological tools such as various videoconferencing platforms (e.g., Microsoft Teams, Blackboard Collaborate, Zoom, and Webex), digital device options (e.g., different kinds of desktops, laptops, tablets, and smartphones) and a wide range of applications and software (e.g. Kahoot, WordWall, Padlet, iMovie, Edmodo, Blogger, Prezi, Canva). PETs should gain experience using these tools to teach and learn OLPE in schools. Therefore, PETs will be able to make OLPE course content efficient and interesting. In addition, interactive activities that will ensure the socio-emotional development of students should be included while preparing the OLPE contents. Moreover, students’ problems, such as access to technology, material, and Internet access, should be minimized by policymakers. Institutional regulations should be applied to ensure student attendance and participation in the course. Overall, even though it seems that the PETs in this study were satisfied with OLPE teaching, after the pandemic, a hybrid education model for OLPE, which merges online and face-to-face training, is suggested.

## Supporting information

S1 File Survey in English version. (DOCX)

S2 File Survey in Turkish version. (DOCX)

S1 Data (SAV)
