# Supplementary material for: Teachers’ perceptions of teaching physical education using online learning during the COVID-19: A quantitative study in Turkey
Source: PLoS One. 2022 Jun 22;17(6):e0269377. doi: 10.1371/journal.pone.0269377 (PMC9216610; doi:10.1371/journal.pone.0269377)
Supplement: S1 File — (DOCX) [file pone.0269377.s001.docx]

**S1 File. Survey in English Version**
